# Vitamin D status among postmenopausal osteoporotic women: a hospital based cross-sectional study from Northern Sri Lanka

**DOI:** 10.1186/s40795-020-00341-y

**Published:** 2020-03-18

**Authors:** Navaneethakrishnan Suganthan, Thirunavukarasu Kumanan, Vithegi Kesavan, Mahalingam Aravinthan, Nadarajah Rajeshkannan

**Affiliations:** 1 Department of Medicine, Faculty of Medicine, University of Jaffna, Jaffna, Sri Lanka; 2 Teaching Hospital, Jaffna, Sri Lanka; 3 Civic Park Medical Centre, Sydney, NSW 2145 Australia

**Keywords:** Vitamin D deficiency, Post-menopausal women, Osteoporosis

## Abstract

**Background:**

Vitamin D deficiency is common among post-menopausal women and it is important to treat vitamin D deficiency to prevent falls and fractures in patients with osteoporosis. Few studies examined the prevalence of vitamin D deficiency in general population of Sri Lanka but no studies to date done among post-menopausal women with osteoporosis in Sri Lanka. This is the first study in Sri Lanka of such kind conducted to evaluate the serum vitamin D levels among postmenopausal women.

**Methods:**

Cross-sectional study was conducted at the Endocrine Unit, Teaching Hospital Jaffna from January to December 2018. During this period 105 postmenopausal women who registered for bone density assessment were recruited to the study. Data collection was done by investigators and blood sample was taken from each participant by registered nurses and total 25-Hydroxy Vitamin D level (25(OH)-Vit D) was measured by competitive immunoassay with enhanced chemiluminiscence technique, levels were categorized and analysis was done using SPSS 26.

**Results:**

Mean age of 105 postmenopausal women was 67.7 with minimum 33 and maximum 84 years. Mean total 25(OH)-Vit D was 27.5 (range11.7–52.5 ng/mL). 25(OH)-Vit D levels were adequate in only 36.2% (95%CI: 27–45), 44% had insufficient levels and deficiency was present in 19% (95%CI: 12–27) of postmenopausal women. Among total study participants 20% were vegetarian, 53, 76.2 and 64.8% were consuming milk, fish and egg respectively and 71.4% reported adequate level of sun exposure (> 30 min/day). Commonly reported vitamin D deficiency symptoms include paraesthesia (57.1%), bone-pain (55.2%), easy fatigability (54.3%), malaise (51.4%), muscle cramps (43.8%) and proximal myopathy (40.0%). Nevertheless, comparison of musculoskeletal symptoms between group with sufficient level and group with insufficient and deficient level showed no significant difference (*P* > 0.05). Among 71 participants (66.7%) who completed bone density assessment, 38% (95%CI: 27–49) showed osteoporosis. Vertebral Z score showed a significant correlation with 25(OH)-Vit D level (r-0.252, P-0.03).

**Conclusion:**

Prevalence of vitamin D deficiency is relatively high among the post-menopausal women with a provisional diagnosis of osteoporosis. It is essential to consider vitamin D supplementation when initiating treatment for osteoporosis. Hence, Vitamin D testing is desirable in decision making to treat or not to treat.

## Background

In recent years the prevalence and the awareness of osteoporosis are increasing and it has been estimated that 200 million of individuals suffer from osteoporosis worldwide [1]. Nevertheless, about 75% of these people represent undiagnosed cases and do not receive appropriate treatment [1, 2]. Bone is remodelled constantly throughout life. Peak bone mass is around the age of 30 years after which rate of bone-resorption is greater than the rate of bone formation. Approximately 3% of cortical bone is replaced each year and 25% of trabecular bone is resorbed and replaced each year [3]. Osteoporosis is defined as bone mineral density less than 2.5 SD below the mean peak value in young adults of the same race and sex (t score of < − 2.5) [4]. Women of all ethnic groups show an additional accelerated phase of bone loss, which occurs for about 10 years after the cessation of ovarian function. Total bone loss in osteoporosis may exceed 30 to 40% [5]. Vitamin D deficiency is a risk factor for fall and fracture among post-menopausal women with osteoporosis [4] and vitamin D deficiency is a pandemic [6] health problem which was attributed for several health problems and well documented in different parts of the globe including Sri Lanka. Moderate level of sun exposure is the major source for Vitamin D as only limited dietary sources are rich in Vitamin D [7]. Anyhow, it is still debatable regarding the cut off level to be treated and most of the evidences for health-related implications of Vitamin D deficiencies are through observational studies and with limited evidences from randomized control trials [8]. Heath problems vary with life cycle and it was well known to link with rickets in paediatric population and osteopenia, osteoporosis and fractures in adults [6, 9].

### Justification

A study of Vitamin D inadequacy among 200 postmenopausal women presenting to Orthopaedics and Gynaecology out-patient departments of Khyber Teaching Hospital in Pakistan, showed high prevalence (59%) of vitamin D deficiency owing to their lack of knowledge, deficient diet, sedentary lifestyle and inadequate sun exposure [10]. In Sri Lanka, as far as to our knowledge, prevalence of Vitamin D status not estimated among postmenopausal women, even though a general prevalence study indicated high prevalence of vitamin D deficiency (57.2%) [11]. Hence, this study was conducted to evaluate serum total 25 hydroxy vitamin D (25(OH)-Vit D) levels, symptoms related to vitamin D inadequacy and factors related to Vitamin D deficiency in postmenopausal women.

## Methodology

### Study design

Hospital based cross -sectional study.

### Place and duration of study

This study was conducted at the Endocrine Unit, Teaching Hospital Jaffna from January 2018 to December 2018.

## Methods

During the study period (January 2018 to December 2018), 105 postmenopausal women who were registered for Dual-Energy X-ray Absorptiometry (DEXA) scan with Endocrine Unit, Teaching Hospital Jaffna were recruited to the study. Women on medications, such as glucocorticoids and anticonvulsants popularly attributed to cause bone loss, individuals on hormone replacement therapy, those with conditions that affect bone metabolism and contributing to osteoporosis, such as multiple myeloma, diseases of the kidney or liver, mal absorption diseases, Paget’s disease, primary hyperparathyroidism, uncontrolled hypo or hyper thyroidism and who has been on vitamin D supplements were excluded from the study.

Data collection was done by investigators using interviewer administered questionnaire and venepuncture was done in aseptic condition. Venous blood sample (10 mL) was taken from each participant by registered nursing officers and sample was analysed by Chemical pathology laboratory at Teaching hospital Jaffna. To maintain uniformity, sample analysis was done by registered medical laboratory technicians at Teaching hospital Jaffna and measured by a competitive immunoassay with enhanced chemiluminiscence detection technique by using Vitros 3600 Immuno Diagnostic system with dedicated reagents from Orthoclinical Diagnostics (Limit of quantitation (LOQ) - 8.00 ng/mL, limit of detection (LOD) - 7.43 ng/mL and reportable range is 8.00–150 ng/mL). Total 25 hydroxy vitamin D was measured and categorized as sufficient (more than 30 ng/mL), insufficient (20–30 ng/mL) and deficient (less than 20 ng/mL) [12]. Same reference range was used previously in Sri Lankan prevalence study [11]. Albumin corrected total plasma calcium level measured among 77 participants and normal reference range for corrected plasma calcium is defined as 2.10 to 2.55 mmol/L.

Study variables were collected by using interviewer administered questionnaire. Data analysis was done using **SPSS** 26 (Statistical Package for Social Sciences). Descriptive statistics such as mean with **SD** (Standard Deviation) were calculated and in addition Chi-square test was used to find the significance between groups. *P* value less than 0.05 was considered as significant. Frequency presented as percentage with 95% **CI** (Confidence Interval). CI was calculated by using WIN PEPI epidemiological software version 11.65 [13]. Bone Mineral Density Assessment was categorized based on the hip bone mineral density using World Health Organisation (WHO) and International Osteoporosis Foundation Dual-energy X-ray absorptiometry (DEXA) assessment diagnostic criteria. T-score is the number of standard deviations below the mean value of the young healthy population [14, 15].

## Results

**Fig. 1:**
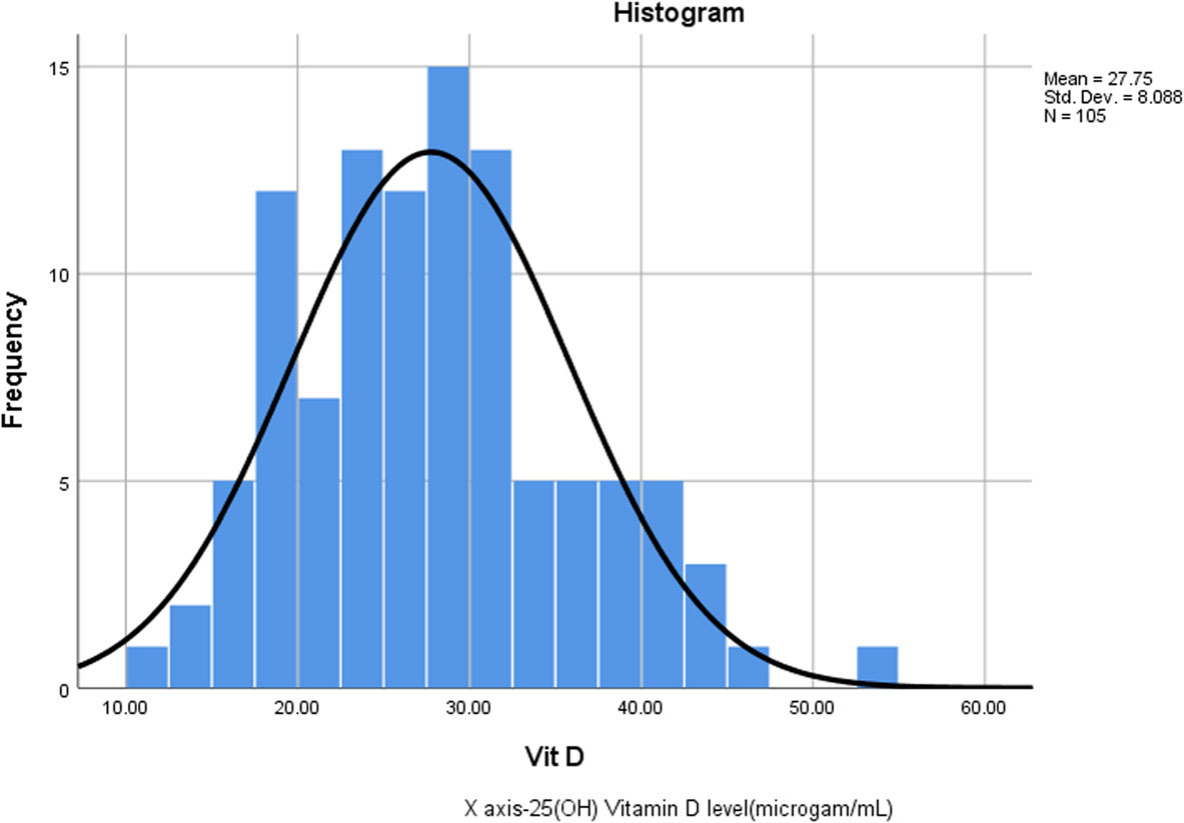
Histogram of the 25(OH)-Vit D levels

Background characteristics: This study included 105 postmenopausal women. Mean age was 67.7 with minimum 33 and maximum 84 years. Twenty-one participants were vegetarian (20%), 24 women (22.9%) reported walking as their main mode of transport, whereas 3.8% used cycling. Furthermore, seventy-five participants (71.4%) reported adequate level of sun exposure (> 30 min/day) and only 2 women reported using sun screen (1.9%). Mean 25(OH)-Vit D level was 27.5 ng /mL with a range of 11.7–52.5 ng/mL (Table 1). The data showed fairly symmetrical distribution (skewness-0.472) (Fig. 1). Albumin corrected plasma Calcium level was measured only among 77 participants and mean calcium level was 2.24 mmol/L with the range of 1.16 mmol/L to 2.54 mmol/L.

**Table 1:** Basic Statistics of Vitamin D level among Participants

Variable	Statistics	Value
Vitamin D Level (ng/mL)	Mean+/_SD	27.8+/_8.1
	Median	27.5
	Skewness	0.472
	Minimum	11.7
	Maximum	52.5
Calcium Level (mmol/L)	Mean+/_SD	2.24+/_0.32
	Median	2.32
	Min	1.16
	Max	2.54
Age	Mean	61.8
	SD	12.2
	Minimum	33
	Maximum	84
Vegetarian	Number (%)	21 (20%)
Walking	Number (%)	24 (22.9%)
Cycling	Number (%)	4 (3.8%)
Adequate level Sun exposure(> 30 min/day)	Number (%)	75 (71.4%)
Using Sun screen	Number (%)	2 (1.9%)

### Prevalence

25(OH)-Vit D levels were adequate only in 36.2% (95%CI: 27.4–45.7) of women. Forty four percent had insufficient levels. Vitamin D deficiency was present in 19% (95%CI: 12.4–27.4) of postmenopausal women. Cumulative prevalence of insufficient and deficient level of 25(OH)-Vit D was 63.8% (95%CI: 54.3–72.6) (Table 2). A positive correlation was observed between age and 25(OH)-Vit D level (Pearson co efficient: r-0.225, *P*-0.021) among study participants.

**Table 2:** Vitamin D Status (Prevalence) among postmenopausal women with suspected osteoporosis

Vitamin D level	Number	Percentage with (95% CI)	Mean	SD
Sufficient Level(*N* = 38) (more than 30 ng/mL)	38	36.2 (27.4–45.7)	36.26	5.43
Insufficient level (*N* = 47) (20–30 ng /mL)	47	44.8 (35.5–54.4)	25.41	2.62
Deficient Level (*N* = 20) (less than20 ng/mL)	20	19 (12.4–27.4)	17.09	2.36
Cumulative Prevalence of deficiency and insufficiency	67	63.8 (54.3–72.6)	27.75	8.09

### Food habits related to vitamin D

Among the 105 participants only 20% were vegetarian and 53% of them consumed milk on average 3 days per week, 76.2% consumed fish on average 2 days per week, 64.8% consumed egg on average 1 day per week (Table 3). Mean 25(OH)-Vit D level among vegetarian group was 28.54 ng/mL, but among non-vegetarian group, level was 27.55 ng/mL(P-0.615).

**Table 3:** Vitamin D rich dietary intake among participants

Vitamin D rich food	Number	Percentage with 95%CI	Number of days in week (mean)
Egg	68	64.8 (55.5–73.4)	1.32
Fish	80	76.2 (67.4–83.6)	2.39
Milk	53	50.5 (41.0–60.0)	3.28
Dairy products	82	78.1 (69.4–85.2)	–

### Symptoms

Commonly reported vitamin D deficiency symptoms are summarised in Table 4. Fifty-eight postmenopausal women reported bone pain paraesthesia (57.1%) followed by bone pain (55.2%), easy fatigability (54.3%), malaise (51.4%), muscle cramps (43.8%) and proximal myopathy (40.0%). Further, comparison of musculoskeletal symptoms between group with sufficient levels of 25(OH)-Vit D and group with insufficient and deficient levels of 25(OH)-Vit D showed statistically not significant results (*P* > 0.05) (Table 5).

**Table 4:** Common musculoskeletal symptoms among postmenopausal symptoms

Symptoms	Number	Percentage with 95% CI
Bone pain	58	55.2 (45.6–64.5)
Easy Fatigability	57	54.3 (44.7–63.6)
Malaise	54	51.4 (41.9–60.9)
Muscle cramps	46	43.8 (34.6–53.4)
Paraesthesia	60	57.1 (47.5–66.4)
Proximal Myopathy	42	40.0 (31.0–49.6)

**Table 5:** Comparison of musculoskeletal symptoms between group with sufficient level 25(−OH) Vit D and group of insufficient and deficient level of 25(−OH) Vit D level

Symptoms	Sufficient level 25(−OH) Vit D	Insufficient & deficient level 25(−OH) Vit D	*P* Value
	No (%)	No (%)	
Bone Pain	18 (31%)	40 (69.0%)	*P* = 0.222
Easy Fatigability	22 (38.6%)	35 (61.4%)	*P* = 0.576
Malaise	16 (29.6%)	38 (70.4%)	*P* = 0.150
Muscle cramps	16 (34.8%)	30 (65.2%)	*P* = 0.791
Paraesthesia	24 (40.0%)	36 (60.0%)	*P* = 0.348
Proximal Myopathy	19 (45.2%)	23 (54.8%)	*P* = 0.115

### Co-morbid conditions

**Fig. 2:**
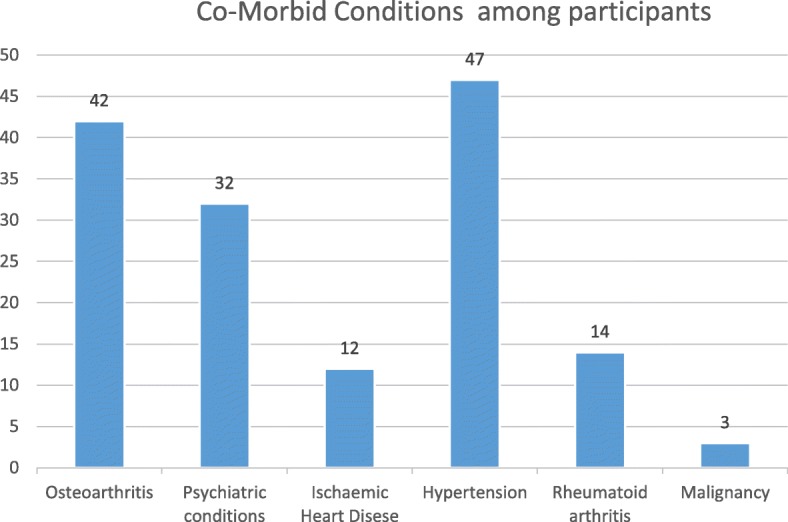
Co Morbid conditions among participants

Comorbid conditions such as hypertension (44.8%), osteoarthritis (41.9%), psychiatric conditions (30.5%), Ischaemic heart disease (IHD) (11.4%), rheumatoid arthritis (3.8%) and malignancy (2.9%) were observed among participants (Fig. 2). None had Type 1 Diabetes Mellitus nor tuberculosis and further analysis showed no association with vitamin D and psychiatric conditions (*P*-0.081) nor with any other comorbid conditions studied (*P* > 0.05) (Table 7).

### Bone density assessment

Among 105 participants 71 completed DEXA scan for bone density assessment (66.7%) (Table 6). Anyhow, postmenopausal women with hip bone T score within osteoporosis range was 38%(CI: 27.3–49.7) and in the osteopenia range was also 38%. It is also worth to note that 7 participants (6.7%) had a prosthesis in situ. Further bivariate analysis showed no significant association between vitamin D deficiency and different categories of bone mineral density (*P* > 0.05). Postmenopausal women with Vertebrae T score in osteoporosis range was high 46 (64.8%) and vertebral Z score showed a significant correlation with 25(OH)-Vit D level (r-0.252, P-0.034). Mean level of Vitamin D among women with osteoporosis was 29.8 ng/mL, whereas mean level among non-osteoporosis was 27.92 ng/mL(P-0.370) (Table 7).

**Table 6:** Bone mineral density assessment results

Category Hip bone mineral density	Number	Percentage with 95% CI
Normal T-score of −1 or higher	17	24% (15.1–34.9)
Low bone mass (osteopenia) T-score between − 1 and − 2.5	27	38% (27.3–49.7)
Osteoporosis T-score of −2.5 or lower	27	38% (27.3–49.7)

**Table 7:** Comparison of Vitamin D level with selected factors

Factors		Number	Mean Vitamin D Level with SD	Statistics
Adequate sun exposure	Yes	75	28.6 + _8.4	T = 1.61, P-0.110
	No	30	25.8 + _7.3	
Walking	Yes	24	27.9 + _9.4	T-0.135 P-0.893
	no	81	27.7_-7.7	
Vegetarian Status	Vegetarian	21	28.5 + _9.4	T-0.5, P-0.615
	Non Vegetarian	84	27.6 + _7.8	
Psychiatric Condition	Yes	32	25.6 + _7.5	T-1.794, P-0.076
	No	73	28.7 + _8.2	
Osteoporosis of Hip	Yes	27	29.8 + _10.4	T-0.903, P-0.370
	No	44	27.9 + _7.6	
Osteoarthritis	Yes	44	27.08 + _8.7	T- -0.721, P-0.473
	No	61	28.2 + _7.7	
Milk	Yes	53	26.9 + _7.8	T- -1.059, P-0.292
	No	52	28.6 + _8.4	
Egg	Yes	68	27.4 + _7.4	T-0.678, P-0.49
	No	37	28.5 + _9.4	
Fish	Yes	80	27.7 + _7.7	T- -0/193, P-0.848
	No	25	28.0 + _9.33	

## Discussion

Current study examined 25(OH)-Vit D levels to determine the prevalence of vitamin D deficiency or insufficiency and also looked into selected associated factors among post-menopausal women with suspected osteoporosis. Bone density assessment was carried out among 77 participants. To the best of our knowledge, this is the first study in the region where Vitamin D status was measured among post-menopausal women even though several studies examined vitamin D level in general population. Gunawardane et.al found that Vitamin D deficiency in Sri Lanka was 57.2% (< 20 ng/mL), vitamin D insufficiency 31% (20–30 ng/mL) and the cumulative prevalence of deficiency & insufficiency was 88.2% [11] and high prevalence was found among young adults (age 18–40) [11]. In the present study, the mean 25(OH)-Vit D concentration was 27.5 ng /mL ±8.09 ng/mL and 19% (95%CI: 12.4–27.4) had vitamin D deficiency (25(OH)- Vit D concentration < 20 ng/mL). Overall, we have found that cumulative deficiency and in- sufficiency of vitamin D was common, 63.8% (95%CI: 54.3–72.6). However, a study among 123 postmenopausal women evaluated in Romania reported 91.9% of them had 25(OH)-Vit D levels below 30 ng/mL [16]. Likewise, a study done in Pakistan among 200 postmenopausal women presented to Orthopaedics and Gynaecology outpatient departments of Khyber Teaching Hospital showed that prevalence of vitamin D deficiency was 59 and 22% had insufficient levels [10]. Almost similar findings revealed in a study from North India which showed vitamin D deficiency among 62% of subjects [17]. Even though, direct comparison among these studies is difficult, relatively low prevalence among our sample could be due to a good exposure to sunlight, a natural source of vitamin D throughout the year as Northern Sri Lanka is located in the tropical region. Historically, most of the requirement of vitamin D is from sun light–induced manufacture of cholecalciferol by skin [7]. Seventy-five of participants (71.4%) reported adequate level of sun exposure (30 min/day). Authors of the study conducted in Pakistan reported that the use of sun protection, wearing purdah and in general women do not go out of their home were possible explanations for low vitamin D levels in their population [10]. However, in Sri Lankan culture, there is no cultural restriction for women to go out from their homes or force to fully cover themselves which support our findings.

Many studies have showed an increasing level of vitamin D deficiency with age [18, 19]. The main reason would be that the elders would have decreased concentrations of precursor of vitamin D3 (7-dehydrocholesterol) that leads to decreased ability to make vitamin D by skin [7]. However, in the present study, 25(OH)-Vit D level showed positive correlation with advancing age (r-0.225, P-0.021). Again the amount of sun exposure is a possible factor contributed to this finding. Young women tend to spend more time indoors with their occupation while a traditional house-wife in Jaffna and the elderly spend more time outdoors. This finding was in par with some previous studies [20, 21] and further a study in Thailand showed young people could have used more sunscreen because of cosmetic reasons [20], however this practice was not observed in this study as only two participants (1.9%) reported to use sunscreen. In terms of dietary sources, common non-fortified food sources include breast milk, cod liver oil, egg yolk, fish such Mackerel (canned), Salmon (canned), Salmon (fresh, farmed), Salmon (fresh, wild), Sardines (canned) Tuna (canned), cat fish, yogurt, margarine, cereals and mushroom [1, 22]. Among 105 participants 53% of them consumed milk on average 3 days per week, 76.2% consumed fish on average 2 days per week, 64.8% consumed egg on average 1 day per week. Nevertheless, no significant difference in 25(OH)- Vit D level was observed between those who consumed vitamin D rich food and those who did not (Table 7). This indicates dietary source not plays a pivotal role.

Vitamin D deficiency symptoms are rather nonspecific which include back pain (non-radiating), arthralgia, proximal muscle weakness, headache, fatigue, altered mood, insomnia and hair loss [23, 24]. In our study 57.1% postmenopausal women reported paraesthesia followed by bone pain (55.2%), easy fatigability (54.3%), malaise (51.4%), muscle cramps (43.8%) and proximal myopathy (40.0%). However, there was no statistical significance observed at 5% level when comparing symptoms among groups with 25(OH)-Vit D deficiency and with adequate levels of 25(OH)-Vit D. This could be due to the fact that the symptoms are non-specific and are common in post-menopausal women even without vitamin D efficiency or might also be associated with other age related co morbid conditions such as osteoarthritis. For example, among 105 participants 41.9% had osteoarthritis and 3.8% had rheumatoid arthritis. The study also investigated the relationship between vitamin D deficiency and menopausal symptoms and concluded that the data is not supportive of vitamin D status association with menopause related symptoms [25].

Vitamin D deficiency reported to be high in prevalence among inpatients with mental illness in previous studies [26, 27]. Thirty percent of samples had psychiatric conditions and mean 25(OH)-Vit D level (25.63 ng/mL) was less among the participants with psychiatric conditions compared to those not having psychiatric conditions (28.68 ng/ml). But this results did not show statistically significant difference (P-0.076).

It is well known fact that prevalence of osteoporosis is common among postmenopausal women and several risk factors implicated for this high prevalence includes vitamin D deficiency. Falls and risk of fractures were well associated with vitamin D deficiency among post-menopausal osteoporosis [4]. Out of 105 post-menopausal women suspected with osteoporosis 71 (66.7%) completed bone density assessment and results revealed osteoporosis was present in 38% (27.3–49.7) and another 38% showed osteopenia. However, Vertebral T score within osteoporosis range was higher (64.8%) and (33.8%) showed osteopenia. Present study failed to show association with different categories of T scores with vitamin D deficiency except Vertebral Z score which showed a significant correlation with 25(OH)-Vit D level (r-0.252, P-0.034). This results could be due to a small size sample and this study was not designed to show this association (not a comparative study). Nevertheless, it is a well-known fact that vitamin D deficiency is more prevalent among post-menopausal women and supplement of vitamin D might prevent of falls and fractures, particularly with people with osteoporosis [4, 28, 29].

The appropriate cut-off level to treat vitamin D deficiency or insufficiency is a dilemma [8]. To maintain minimum required 25(OH)-Vit D level (30 to 32 ng /mL) requires 2200 to 3000 IU/day from all available resources including sun exposure, food and supplements [30–32]. Further, age specific recommendations suggest 200 IU of vitamin D daily from birth to age 50, 400 IU/day for age 51 to 70 years, and 600 IU/day for those aged 70 years and above [33, 34] This recommendation presume that usual sources of vitamin D such as sun exposure and food are not adequate [31, 32]. It is an observation that vitamin D supplements for all post-menopausal women may lead to hypervitaminosis D. Still, supplementation with vitamin D for post-menopausal women with vitamin deficiency is beneficial in preventing osteoporosis especially to prevent complications of fall and fracture [11, 16]. Since high prevalence of vitamin D deficiency among post-menopausal women with suspected osteoporosis has been shown by this study, it emphasizes the fact that early screening for suboptimal 25(OH)-Vit D level among the above group is crucial to prevent osteoporotic fractures and falls.

### Limitations

The strengths of this study are that this was the first study in Sri Lanka specifically examined the prevalence of vitamin D deficiency among post-menopausal women with suspected osteoporosis and explored some protective factors like sun exposure. 25(OH)-Vit D level measured by competitive immunoassay with enhanced chemiluminiscence technique method which is one of the standard clinical laboratory methods. However, some limitations of the study includes that we did not obtain information about some anthropological measurements such as **BMI** (Body Mass Index); physical activity; socioeconomic status and the influence of seasonal effects and climatic changes on vitamin D deficiency. Calcium level measurement completed only among 77 participants due financial and social reasons. Furthermore, sample size estimated only with the aim of estimating prevalence but validity of the study would have been improved if we had an estimated sample size for sub analysis. Some participants (33.3%) did not complete bone density assessments which could be the reason for some factors not showing statistically significant association, even though some relationship observed in psychiatric condition, sun exposure, z/t scores of vertebral DEXA and 25(OH)-Vit D level.

## Conclusions

As this study demonstrated relatively high prevalence of vitamin D deficiency among post-menopausal women with suspected osteoporosis, treatment of vitamin D deficiency with supplementation is essential to prevent fractures in whom vitamin D through dietary sources and sunlight exposure are not sufficient. In addition, it is essential to consider vitamin D supplementation when initiating treatment for osteoporosis in particular the bisphosphonate therapy. The authors recommend routine testing of vitamin D in postmenopausal women of this population in order to make concrete decisions to initiate Vitamin D supplementation as a routine.

## Data Availability

Data can be provided on request from NR or NS.

## Abbreviations

25(OH)-Vit D: 25 hydroxy vitamin D
BMI: Body Mass Index
CI: Confidence Interval
DEXA: Dual-Energy X-ray Absorptiometry
IHD: Ischaemic Heart Disease,
SD: Standard Deviation
SPSS: Statistical Package for the Social Sciences
WHO: World Health Organisation
